# Targeting JNK pathway promotes human hematopoietic stem cell expansion

**DOI:** 10.1038/s41421-018-0072-8

**Published:** 2019-01-08

**Authors:** Xiong Xiao, Weifeng Lai, Huangfan Xie, Yang Liu, Weijie Guo, Yifang Liu, Yu Li, Yuanjun Li, Jingliang Zhang, Wenhan Chen, Minhui Shi, Lijun Shang, Ming Yin, Chengyan Wang, Hongkui Deng

**Affiliations:** 1 State Key Laboratory of Chemical Oncogenomics, School of Chemical Biology & Biotechnology, Peking University Shenzhen Graduate School, Shenzhen, Guangdong, 518055 China; 2 The MOE Key Laboratory of Cell Proliferation and Differentiation, College of Life Sciences, Peking-Tsinghua Center for Life Sciences, Peking University, Beijing, 100871 China; 3 Department of Cell Biology, School of Basic Medical Sciences, Peking University Stem Cell Research Center, Center for Molecular and Translational Medicine, State Key Laboratory of Natural and Biomimetic Drugs, Peking University Health Science Center, Beijing, 100191 China; 4 Joint Graduate Program of Peking-Tsinghua-NIBS, School of Life Sciences, Tsinghua University, Beijing, 100084 China; 5 Beijing Vitalstar Biotechnology, Beijing, 100012 China

**Keywords:** Haematopoietic stem cells, Extracellular signalling molecules

## Abstract

The limited number of human hematopoietic stem cells (HSCs) has restrained their widespread clinical application. Despite great efforts in recent years, the in vitro expansion of HSCs remains a challenge due to incomplete understanding of the signaling networks underlying HSC self-renewal. Here, we show that culturing human cord blood (CB) CD34^+^ cells with JNK-IN-8, an inhibitor of the JNK signaling pathway, can enhance the self-renewal of HSCs with a 3.88-fold increase in cell number. These cultured CD34^+^ cells repopulated recipient mice for 21 weeks and can form secondary engraftment that lasted for more than 21 weeks. Knockdown of *c-Jun*, a major downstream target in the JNK pathway, promoted the expansion of hematopoietic stem and progenitor cells (HSPCs). Our findings demonstrate a critical role of the JNK pathway in regulating HSC expansion, provide new insights into HSC self-renewal mechanism, and may lead to improved clinical application of HSCs.

## Introduction

Hematopoietic stem cell transplantation is a well-established stem-cell therapy for leukemia and other high-risk blood diseases with more than 40,000 applications worldwide annually^1,2^. Despite this success, most patients are excluded from the transplant procedure due to a lack of matched donors^3^. Besides bone marrow (BM) and mobilized peripheral blood (mPB), cord blood (CB) is an alternative source of allogeneic HSC transplantation because the great number of banked CB units can help find an appropriate graft. However, the insufficient number of HSCs in a single CB unit limits their clinical application^4^. Thus, there is a pressing demand to expand HSCs in vitro for improving the clinical efficacy of HSC transplantation.

The major question on in vitro expansion of HSCs is how to promote cell proliferation while retaining their long-term repopulating ability. Earlier studies showed that in vitro HSC expansion with cytokines induced only progenitor cell proliferation and HSC differentiation^5^. Subsequent studies showed that manipulations of Wnt and mTOR signaling pathways suppress differentiation and help to maintain the repopulating HSCs^6,7^. Recently, StemRegenin1 (SR1), an aryl hydrocarbon receptor (AhR) signaling suppressor, and UM171, a pyrimidoindole derivative, were found to enhance significantly the in vitro expansion of long-term repopulating HSCs^8,9^. However, SR1-treatment caused the delayed T-cell recovery^10^, and the mechanism of UM171-induced expansion remains unclear^9,11^. Further investigation is therefore required to establish a new protocol for HSC expansion.

In this study, we focused on the JNK pathway and evaluated the effect of the related compounds on HSC expansion, and found that JNK-IN-8, a JNK inhibitor, can expand human CB HSCs with long-term repopulating capacity. Our results suggest a role of JNK pathway in HSC self-renewal regulation and offer a novel promising approach to further improve HSC expansion.

## Results

### JNK signaling pathway inhibition was identified to expand HSPCs in vitro

**Fig. 1:**
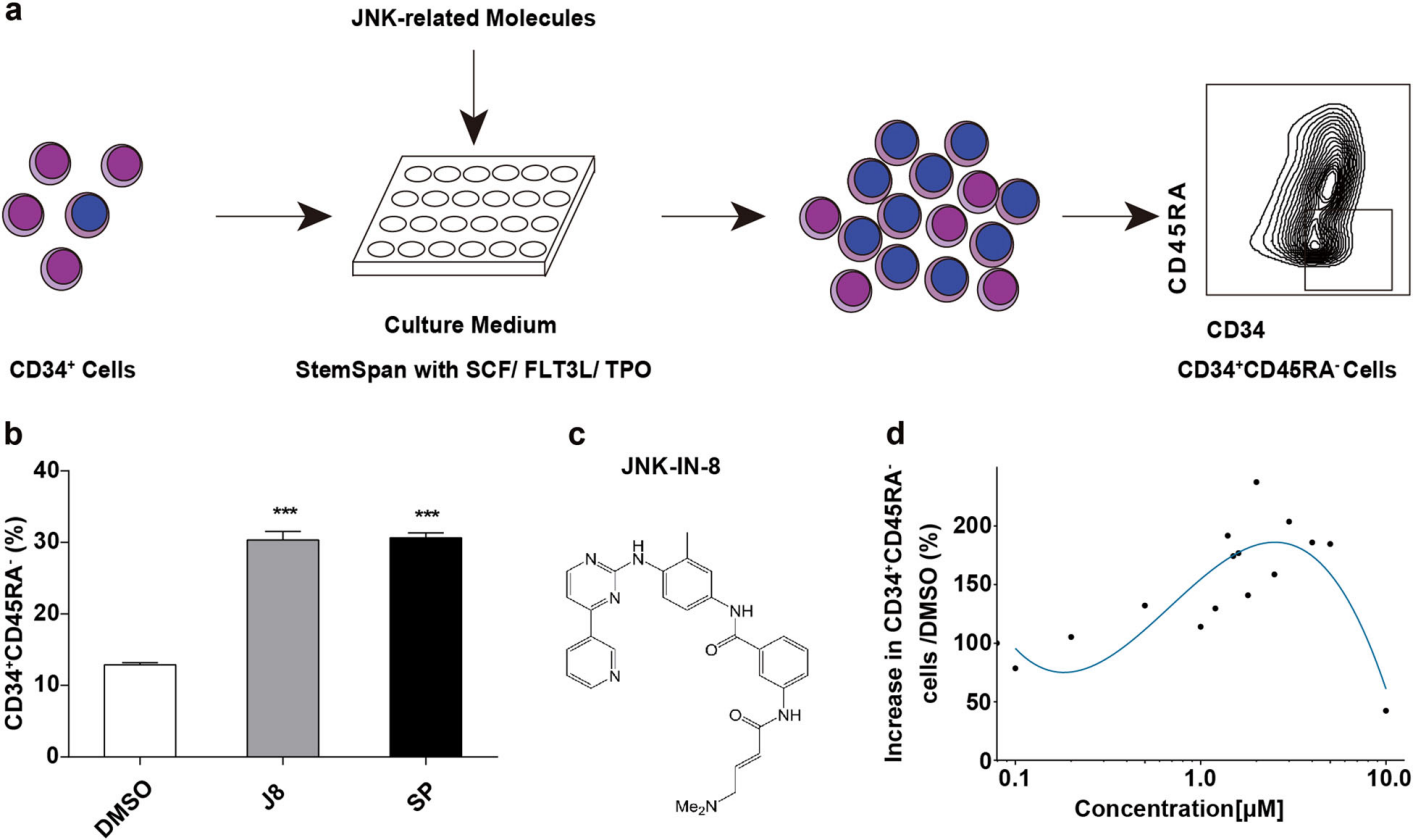
JNK-IN-8 promotes HSPC expansion in vitro. **a** Experimental schematic for the evaluation of JNK inhibitors on HSC expansion. CD34^+^ cells were cultured with StemSpan SFEM II medium supplemented with SCF, TPO, FLT3L in the presence of JNK-related molecules for 7 days, then the total cell expansion and CD34^+^CD45RA^-^ cell frequency was determined. **b** Frequency of CD34^+^CD45RA^-^ cell subsets in 7-day cultures of CD34^+^ cells supplemented with two representative JNK inhibitors including JNK-IN-8 (2 μM) and SP600125 (5 μM) (*n* = 3 experiments). **c** Chemical structure for JNK-IN-8 (hereafter called J8). **d** The increasing fold of CD34^+^CD45RA^-^ subset frequency compared to DMSO after 10-day culture of CD34^+^ cells with different concentration of J8 (This data was drawn by R). See also to Supplementary Table S1. All data shown as mean values ± SD. Statistical significance was assessed using unpaired *t* test, where ***p < 0.001. See also Supplementary Fig. S1

Since recent reports indicate that self-renewing tissue-resident macrophages and embryonic stem cells share the same regulatory network for self-renewal^12^, we hypothesized that common signals may also regulate the self-renewal of pluripotent stem cells and of HSCs. One of the important pathways that regulate the self-renewal of pluripotent stem cell but has not been described in HSC expansion, is the JNK signaling pathway^13–15^, which also participates in the development of leukemogenesis^16,17^. Thus, we tested a panel of JNK-related small molecules for their ability to stimulate the expansion of human CD34^+^CD45RA^−^ CB cells^18^, which are enriched in HSCs (Fig. 1a)^19,20^. We found that the JNK inhibitor, JNK-IN-8, or SP600125, when added in the medium of a 7-day culture, significantly increased the frequency and number of CD34^+^CD45RA^−^ cells compared with DMSO-treated control cells (Fig. 1b; Supplementary Fig. S1a). For JNK-IN-8 (Fig. 1c) is a more specific JNK inhibitor than SP600125, we further studied the effects of JNK-IN-8 on the expansion of HSCs in vitro and in vivo^18^.

**Fig. 2:**
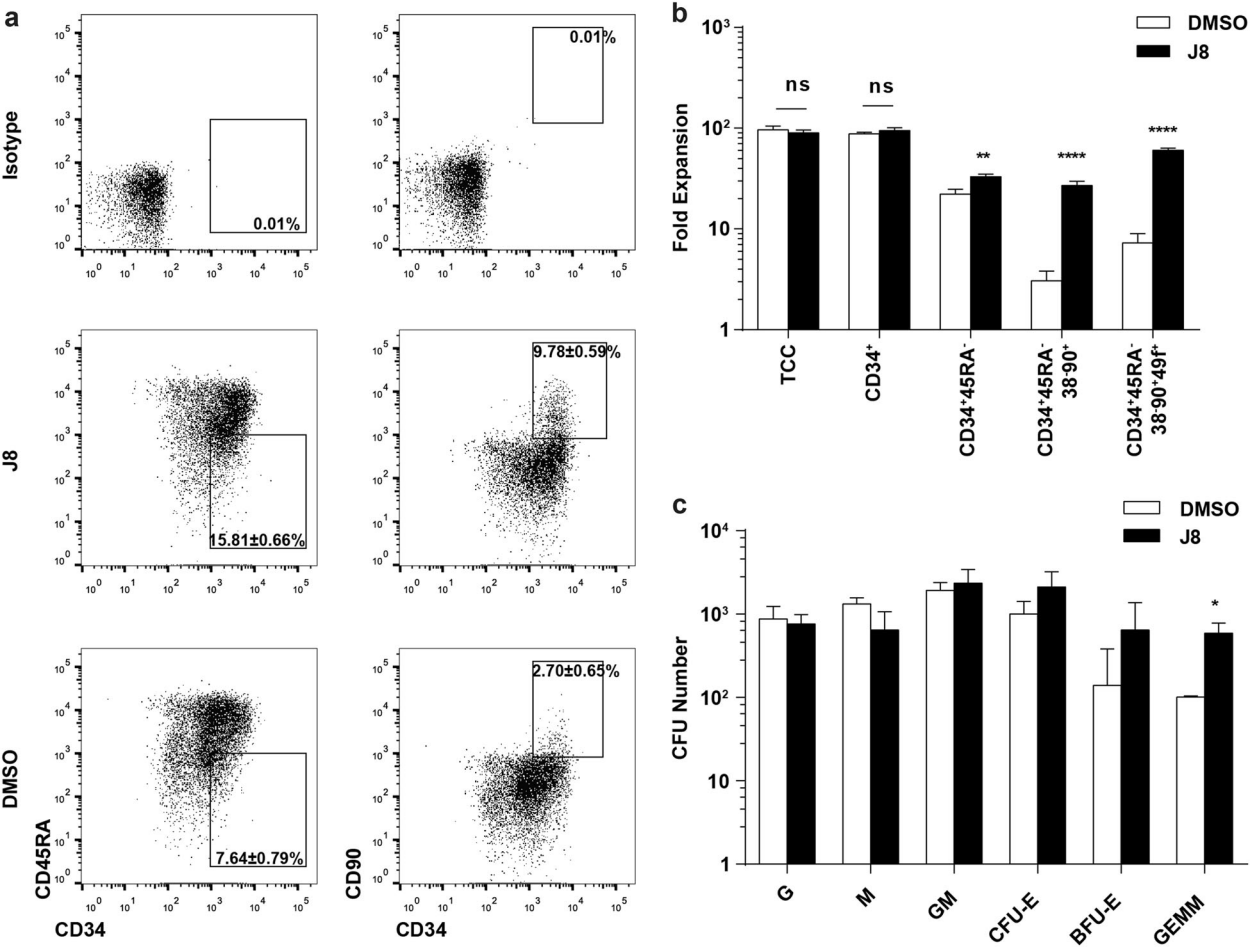
The effects of JNK-IN-8 on cell expansion. **a** Representative flow plots of phenotypically defined cell subsets after 10-day culture of 10,000 fresh CD34^+^ cells supplemented with DMSO or J8 (2 μM) (*n* = 3 experiments). **b** Fold expansion of indicated phenotypically defined cell population after 10-day culture of 10,000 fresh CD34^+^ cells supplemented with DMSO or J8 (2 μM) (*n* = 3 experiments). TCC, total cell count. **c** CFU number of progenies from 1,000 day 0 CD34^+^ cells after 10-day culture supplemented with DMSO or J8 (2 μM) (*n* = 3 experiments). G, CFU-granulocyte; M, CFU-macrophage; GM, CFU-granulocyte and macrophage; CFU-E CFU-erythrocyte; BFU-E, erythroid burst-forming unit; GEMM, CFU-granulocyte, erythroid, macrophage, and megakaryocyte. All data shown as mean values ± SD. Statistical significance was assessed using unpaired *t* test, where *p < 0.05, **p < 0.01, and ****p < 0.0001; ns, not significant. See also Supplementary Fig. S1 and Table S2

To optimize the use of JNK-IN-8 for improving HSC expansion, we tested different dosage of JNK-IN-8 for the expansion of CD34^+^CD45RA^−^ CB cells. The highest expansion level of these cells was observed with 2 μM JNK-IN-8 treatment (Fig. 1d; Supplementary Table S1). We also found that the treatment of JNK-IN-8 supplied with cytokines for 10 days led to an enhanced expansion of CD34^+^CD45RA^−^CD38^−^CD90^+^ cells, which is eightfold significantly higher than the control group (cytokines plus 0.01% of DMSO; Supplementary Fig. S1b). In addition, we found that the CD34^+^ cells exhibited high frequency of CD34^+^CD45RA^−^ cells (15.81 ± 0.66% vs. 7.64 ± 0.79% in the control group) and CD34^+^CD90^+^ cells (9.78 ± 0.59% vs. 2.70 ± 0.65% in the control group) in JNK-IN-8-treated group (Fig. 2a). Finally, we found that the JNK-IN-8 treatment greatly expanded HSPC subsets CD34^+^CD45RA^−^, CD34^+^CD38^−^CD45RA^−^CD90^+^, and CD34^+^CD38^−^CD45RA^−^CD90^+^CD49f^+^ cells (Fig. 2b; Supplementary Table S1). These results indicate that JNK-IN-8 can efficiently expand HSPCs in vitro.

To determine whether the effect of JNK-IN-8 in HSC expansion was reversible, we washed the JNK-IN-8-treated CD34^+^ cells at day 7 and cultured them with cytokine-only medium for an additional 5 days. This led to about 70%-decrease of proportion of CD34^+^CD45RA^−^ cells, suggesting that JNK-IN-8 is indispensable for the whole process of HSC expansion (Supplementary Fig. S1c). We also observed that hematopoietic cytokines including SCF, FLT3L, and TPO are required to enhance the function of JNK-IN-8 (Supplementary Fig. S1d). Finally, cell division kinetic analysis by carboxyfluorescein succinimidyl ester (CFSE) staining showed that JNK-IN-8 did not significantly affect cell division rate of the phenotypically primitive HSPC populations (Supplementary Fig. S1e).

To further define the multipotency of JNK-IN-8-expanded cells, we performed the colony-forming unit (CFU) assay. We found that JNK-IN-8 can also increase the colony-forming unit of granulocyte, erythrocyte, macrophage, and megakaryocyte (CFU-GEMM) by about four folds, on day 10 (Fig. 2c), while the number of other types of CFUs, including the colony-forming units of granulocyte (CFU-Gs), macrophage (CFU-Ms), erythrocyte (CFU-Es), granulocyte and macrophage (CFU-GMs), or burst-forming units of erythrocytes (BFU-Es) were not significantly influenced by JNK-IN-8 treatment (Fig. 2c; Supplementary Table S2). Together, these results suggest that JNK-IN-8 promoted expansion of multipotent HSPCs.

### JNK-IN-8 expanded HSCs with long-term repopulating capacity

**Fig. 3:**
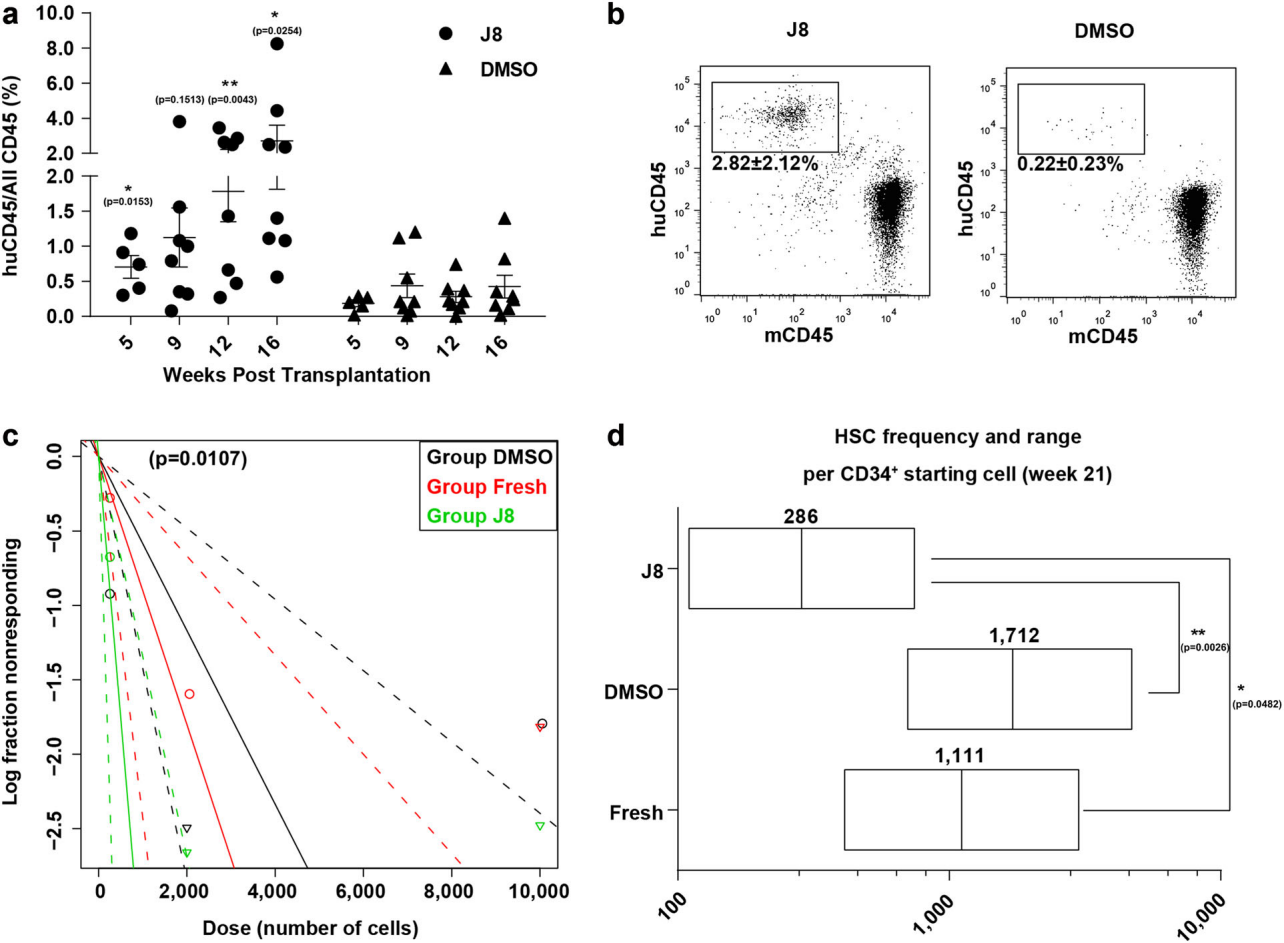
JNK-IN-8 promotes expansion of HSCs in primary recipients. **a** Human CD45^+^ engraftment level in the PB of recipients transplanted with DMSO or J8-expanded 10,000 day 0 CD34^+^ cells on 5, 9, 12, 16 weeks posttransplantation (*n* = 2 independent experiments). Statistics significance between DMSO and J8 group on 5, 9, 12, 16 weeks was assessed by multiple *t* test respectively, where *p < 0.05, **p < 0.01. **b** Representative flow plots of engraftment of J8-expanded and DMSO-expanded CD34^+^ cells in mouse recipients’ PB 16 weeks after transplantation. Progenies of 10,000 day 0 CD34^+^ cells after 10-day culture with DMSO or J8 were transplanted per mouse. **c** HSC frequencies of fresh CD34^+^ cells, J8, and DMSO cultured cells determined by LDA, calculated by ELDA software. As for overall test for differences in stem cell frequencies between any of the groups, *p* = 0.0055. The frequency of more than 1% human chimerism (human CD45 compared with all CD45) in the BM was regarded as positive response. **d** HSC frequencies presented as 1/fresh CD34^+^ cells equivalent for each condition from (c), the required confidence interval was 95%. See Supplementary Table S3A. All data shown as mean values ± SD. Statistical significance was assessed using unpaired *t* test, where *p < 0.05, and **p < 0.01. See also Supplementary Fig. S2

To determine whether JNK-IN-8 treatment could enhance the engrafting ability CD34^+^ cells, we injected 10,000 JNK-IN-8/DMSO-treated CB CD34^+^ progeny cells after 10-day culture into immunodeficient mice and then quantified the reconstituted human CD45^+^ cell frequency in peripheral blood (PB) of the recipient mice after 5, 9, 12, and 16 weeks. We found that the JNK-IN-8-expanded cells showed a sustainably increased engraftment rate in the PB at all these times, as compared with control cells (Fig. 3a). Specifically, at 16 weeks posttransplantation, the JNK-IN-8-expanded cells showed higher percentage of human engraftment in the PB (Fig. 3b) than the controls. These data indicate that the JNK-IN-8 treatment greatly increased the engrafting ability of HSCs.

To detect the frequency (adjusted to day 0 CD34^+^ cell number) of the HSCs after a 10-day JNK-IN-8 treatment, we performed limiting dilution assay (LDA) by transplanting progenies of 200/2,000/10,000 starting cells into immune-deficient NPG mice. The HSC frequency, when analyzed at 21 weeks posttransplantation, was 1/1,111 for the original fresh CD34^+^ cells and 1/1,712 for DMSO control cells but was significantly higher at 1/286 for the JNK-IN-8-expanded cells. This represents a 3.88-fold expansion of the repopulating HSCs in culture with JNK-IN-8 (Fig. 2c, d; for more details see Supplementary Table S3A).

To evaluate whether the JNK-IN-8-treated CD34^+^ cells retain normal lineage differentiation potential, we analyzed the hematopoietic output in the BM of recipient mice at 21 weeks posttransplantation. We found that the JNK-IN-8-treated cells exhibited normal lineage repopulation potential compared with control cells, giving rise to myeloid (CD33^+^), and lymphoid (CD19^+^) cells in vivo (Supplementary Fig. S2a, b). These results indicate that human engrafts of the JNK-IN-8-expanded cells are comprised primarily of lymphoid cells and myeloid cells, similar to the freshly isolated cells and the DMSO control cells (Supplementary Fig. S2a, b).

**Fig. 4:**
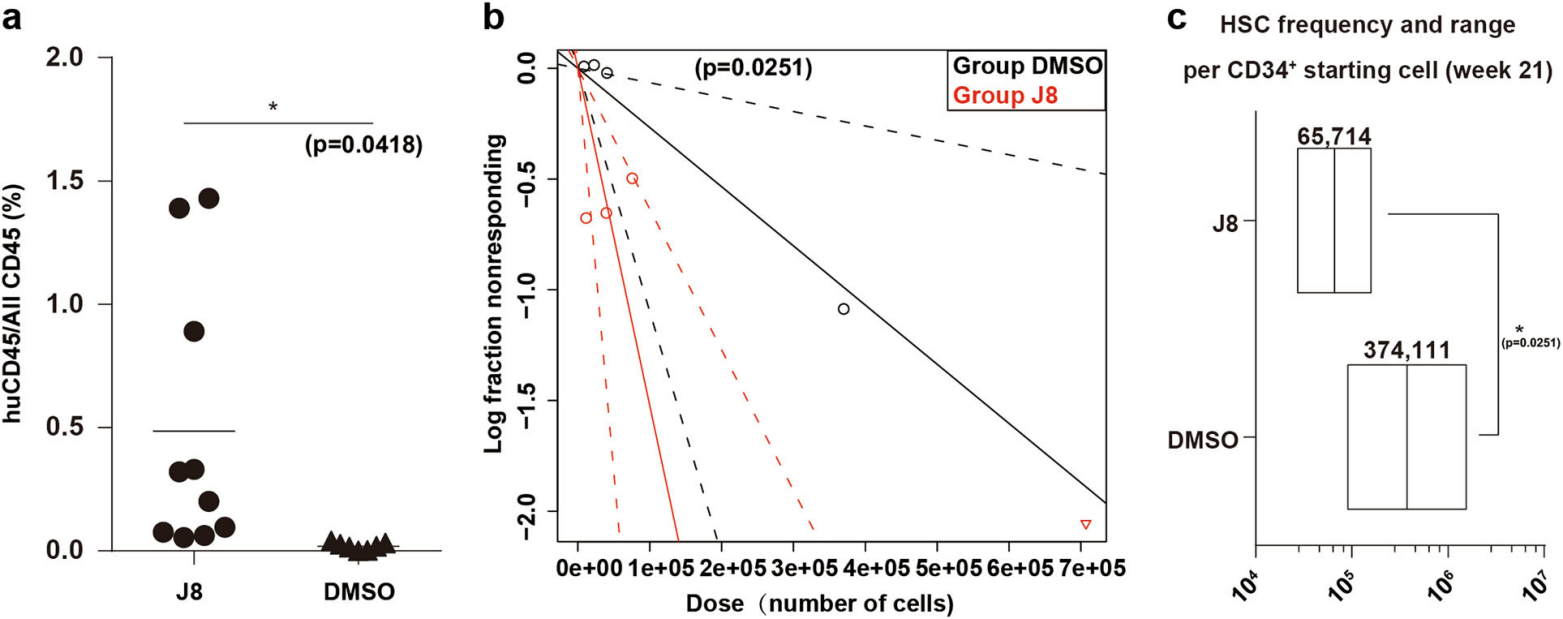
JNK-IN-8-expanded HSCs repopulate secondary recipients. **a** Secondary engraftment in mouse recipients’ PB 21 weeks after transplantation of 1 × 10^7^ cells from BM of the primary recipients injected with DMSO or J8-expanded 10,000 day 0 CD34^+^ cells 21 weeks after transplantation. (*n* = 10 mouse from two independent experiments per group, *p = 0.0418 by two-tailed unpaired *t* test.) **b** HSC frequency in secondary recipient of J8 or DMSO-expanded cells calculated by ELDA software. More than 1% human CD45 engraftment in the BM was regarded as positive. As for overall test for differences in stem cell frequencies between any of the groups, *p* = 0.0251. **c** HSC frequencies presented as 1/fresh CD34^+^ cells equivalent for each condition from (b), the required confidence interval was 95%. More than 1% human CD45 engraftment in the BM was regarded as positive. See also Supplementary Table S3B. All data shown as mean values ± SD. Statistical significance was assessed using unpaired *t* test, where *p < 0.05

To determine whether the JNK-IN-8-expanded cells can sustain serial engraftment, we performed the secondary transplantation with the same dosage of BM cells isolated from primary recipient mice injected with JNK-IN-8 or DMSO cultured cells at 21 weeks posttransplantation. We found that these cells successfully established human engraftment in secondary recipients at 21 weeks posttransplantation, at levels that were significantly higher than the DMSO control group in PB (Fig. 4a), suggesting expansion of long-term HSCs (LT-HSCs) in JNK-IN-8-treated cells. The frequency of HSCs in JNK-IN-8-treated cells (1/65,714), as examined by LDA assay was 5.69 times of the DMSO control (1/374,111) (Fig. 3b, c; for more details see Supplementary Table S3B). These results demonstrate that the JNK-IN-8-expanded cells retain the ability to form multilineage of blood cells and exhibit long-term secondary engrafting capacity.

### JNK-IN-8 promoted HSPC expansion through c-Jun

**Fig. 5:**
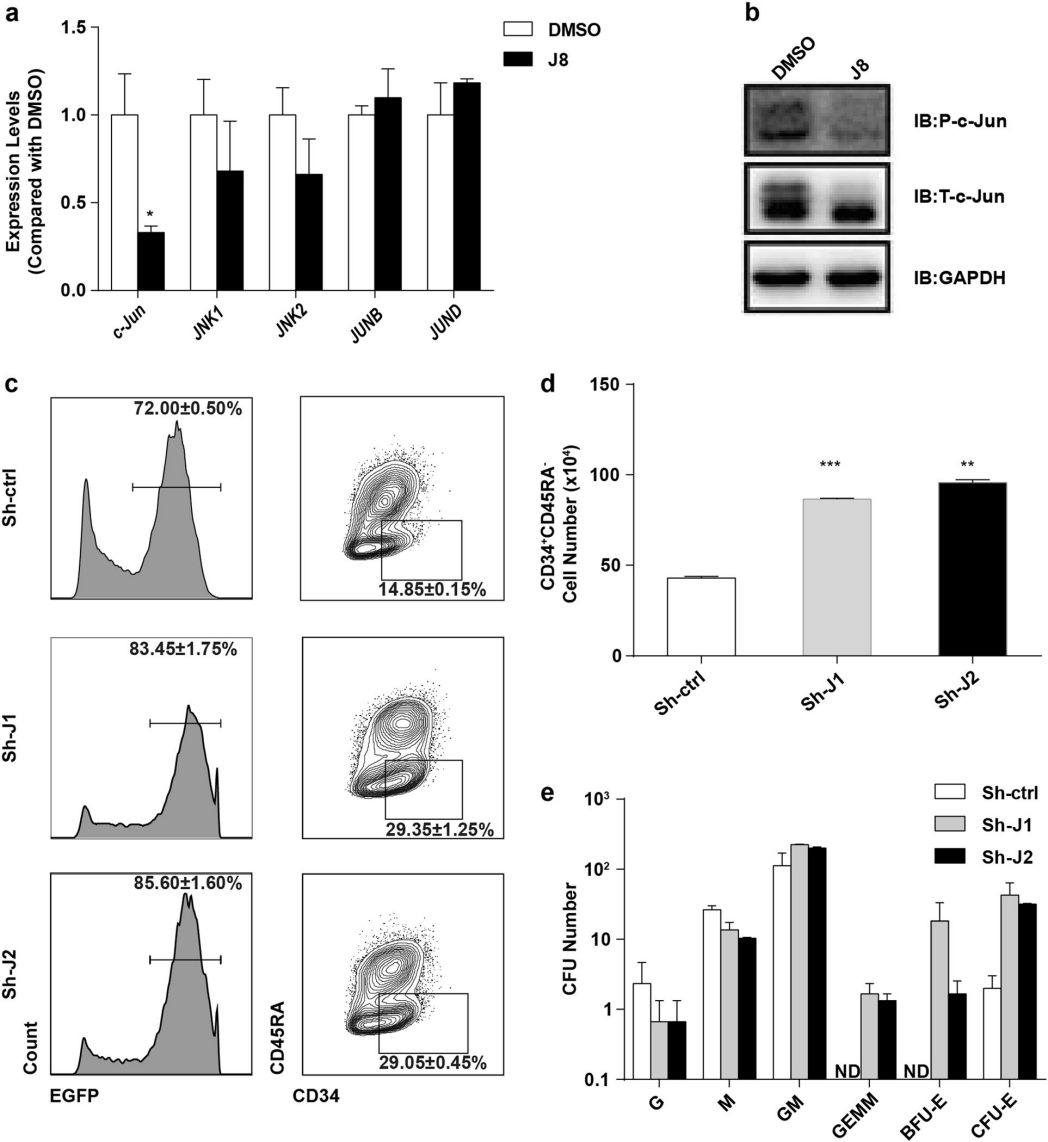
JNK-IN-8-induced CD34^+^ cell expansion acts by inhibiting c-Jun. **a** Relative mRNA expression of indicated JNK-related genes on day 5, CD34^+^ cells cultured with DMSO or J8 (*n* = 3 experiments). **b** Western blot analysis of inhibition of phosphorylated c-Jun for DMSO and J8-treated CD34^+^ cells following serum stimulation for 30 min. **c** Representative flow cytometry profiles of CD34^+^ cells 5 days after transduction with lentivirus expressing shRNAs targeting *c-Jun* or scrambled shRNAs (*n* = 3 experiments). **d** Total cell number of CD34^+^CD45RA^-^ cell population in indicated cultured CD34^+^ cells on day 5 posttransfection (*n* = 3 experiments). **e** CFU numbers of 5, 000 cells on day 5 after transduction with lentivirus expressing shRNAs targeting *c-Jun* or scrambled shRNA (*n* = 3 experiments). G, CFU-granulocyte; M, CFU-macrophage; GM, CFU-granulocyte and macrophage; CFU-E, CFU-erythrocyte; BFU-E, erythroid burst-forming units; GEMM, CFU-granulocyte, erythroid, macrophage, and megakaryocyte. See also Table S4A. Sh-ctrl, CD34^+^ cells transfected with scrambled shRNA; Sh-J1, CD34^+^ cells transfected with 1# c-Jun shRNA; Sh-J2, CD34^+^ cells transfected with 2# c-Jun shRNA. All data shown as mean values ± SD. Statistical significance was assessed using unpaired *t-*test, where *p < 0.05; **p < 0.01, and ***p < 0.001; ND, not detected. See also Supplementary Fig. S3

To determine the mechanism by which JNK-IN-8 promoted human HSC expansion, we compared the expression of genes involved in JNK signaling pathway in the JNK-IN-8-expanded versus the control CD34^+^ cells. As was the RNA-seq data shown, among the JNK pathway genes, the expression of *JUN* changed most significantly in JNK-IN-8-expanded cells, followed by *STAT4, MAP1B*, and *GCKR*, etc (Supplementary Fig. S3a, b). Among the tested genes, the expression of *JUN* was significantly downregulated about five times in JNK-IN-8-expanded cells compared with DMSO-treated cells, while the expression of other JNK downstream genes did not show significant change (Supplementary Fig. S3a, b). We further confirmed the reduction of the mRNA expression of *c-Jun* by JNK-IN-8 treatment using quantitative real-time PCR assay; the expression of major JNK signaling-related genes, like *JNK1*, *JNK2*, *JUNB,* and *JUND* were not affected after JNK-IN-8 treatment (Fig. 5a)^21^. Moreover, as the western blot assay showed, after the JNK-IN-8 treatment, total c-Jun was slightly reduced (Fig. 5b; Supplementary Fig. S3c), and the phosphorylation of c-Jun protein was significantly decreased by nearly 50% (Fig. 5b; Supplementary Fig. S3d). Together, these data suggest that JNK-IN-8 inhibits JNK pathway via c-Jun.

To understand whether c-Jun is indeed a crucial mediator for HSC expansion, we targeted *c-Jun* by transducing CD34^+^ cells with lentiviral vector carrying short hairpin-mediated RNAs (shRNAs) and enhanced green fluorescent protein (EGFP) (Supplementary Fig. S3e). The control CD34^+^ cells were transduced with lentivirus that expressed scrambled shRNA and EGFP. We observed that knockdown of *c-Jun* led to nearly 70% decrease in its mRNA expression level (Supplementary Fig. S3f). These *c-Jun*-deficient cells showed an increased CD34^+^CD45RA^−^ cell frequency and number at day 5 posttransduction, which is more than twofold higher than the control (Fig. 5c, d). The knockdown of *c-Jun* also led to the expansion of multipotent progenitors with more CFU-GEMMs, and an increased number of BFU-Es and CFU-Es compared with scrambled shRNA control (Fig. 5e). Other CFUs, like CFU-Gs, CFU-Ms, and CFU-GMs, showed no significant difference between the *c-Jun* knockdown and control groups (Fig. 5e; Supplementary Table S4A). Furthermore, the *c-Jun* shRNA-transduced CD34^+^ cells showed significantly enhanced engraftment efficiency as compared with the control (Supplementary Fig. S3g; Supplementary Table S4B). Taken together, these results suggest that c-Jun inhibition may be a key mechanism for the JNK-IN-8-mediated expansion of the HSCs.

## Discussion

In this study, we discovered that JNK is a novel and crucial signal pathway to regulate the expansion of human HSCs. Inhibition of JNK pathway with chemical compound of JNK-IN-8 or by genetic manipulation can enhance the expansion of human HSCs. Moreover, JNK-IN-8-expanded HSCs can sustain long-term repopulating capacity and multipotent potential with primary engraftment for 21 weeks and secondary engraftment for more than 21 weeks. Interestingly, a recent study that ectopic expression of miR-125a augmented CD34^+^ CB HSC serial engraftment showed that miR-125a-overexpressed CD34^+^ cells possessed significant downregulation of JNK pathway effectors^22^. Therefore, together with our data, JNK signal may be an important signaling pathway with good potential in regulating human HSC expansion, which deserves further study.

Our study pinpointed c-Jun as a pivotal downstream effector for JNK-IN-8-mediated human HSC expansion. Interestingly, among the JNK-signal related genes, only the expression of *c-Jun* was identified to be changed mostly after JNK-IN-8 was added in the culture, which led to a speculation that the expansion of HSCs with JNK-IN-8 might be through targeting c-Jun. c-Jun is a component of AP-1 complex composed of many subunits like Fos, FosB, JunB, and JunD^23^. Previous study showed that c-Jun promoted myeloid differentiation by enhancing PU.1 or M-CSF transcription^24,25^, suggests that downregulation of c-Jun can promote HSC self-renewal and expansion by preventing HSC from rapid differentiation. Although there has been some evidence in mice that c-Jun-related transcription factors affect HSC self-renewal and differentiation^16,17,26–28^, whether c-Jun participates in human HSC expansion has not been elucidated. Our data show that downregulation of c-Jun by JNK-IN-8 or shRNA knockdown increased the number of human multipotent progenitors and engraftable HSCs. Therefore, our findings defined, for the first time, c-Jun as a critical target for human HSC expansion, which extends the current understanding of HSC self-renewal regulation.

In summary, our study demonstrates that targeting JNK signaling via c-Jun can promote human HSC expansion. Additional studies are needed to determine whether JNK inhibition can exert synergistic effects on promoting HSC self-renewal with SR1, UM171, or other HSC self-renewal modulators such as most recently identified PPARγ antagonist GW9662 (ref. ^29^) or HDAC5 inhibitor LMK235 (ref. ^30^). Finally, future studies of the involvement of the JNK pathway in HSC proliferation may yield new clues and strategies to facilitate the expansion of HSCs and may lead to further improvement of the clinical application of human HSCs.

## Materials and methods

### Cord blood

This study was approved by the Institute of Review Board in Peking University (IRB 00001052-15087) and conducted according to the approved protocol. Samples were collected from consenting donors according to ethically approved procedures at China–Japanese Friendship Hospital.

### Mice

All the animal procedures were performed abiding by Animal Protection Guidelines of Peking University, China. All the mouse experiments were approved by the Institutional Animal Care and Use Committee of Peking University. All of the mice for transplantation were NOD-Prkdcscid Il2rgtm1/Vst (NPG) mice (Stock No.: VS-AM-001) purchased from Beijing Vitalstar Biotechnology, ranging from 8 to 12 weeks of age.

### Collection and isolation of human CD34^+^ hematopoietic stem/progenitor cells from cord blood

Human CD34^+^ CB cells were isolated using CD34 MicroBead Kit (Miltenyi Biotec, Cat: 130-046-703) according to the manufacturer’s instructions. Briefly, CB unit was diluted by sterile PBS to about 360 ml and aliquoted gently to human lymphocyte separation medium (DRKEWE, Cat: DKW-KLSH-0100) in 50 ml tube, ending with 35 ml CB dilution, and 15 ml lymphocyte separation liquid in each tube. The tubes were centrifuged at 1,500 rpm for 20 min. The monocytes were collected from the middle layer of the liquid and washed twice with PBS (CORNING, Cat: 21–040-CV) through centrifuging at 1,600rpm for 10 min. Then they were stained with equivalent CD34^+^ MicroBeads and FcR Blocking Reagent at 4 °C for 30 min. Incubated cells were washed with PBS for three times. Then, the cell suspension was loaded onto a MACS^®^ Column, which was placed on the magnetic field of a MACS separator. After removing the column from the magnetic field, the magnetically retained CD34^+^ cells were eluted as the positively selected cell fraction into a new tube.

### Human CD34^+^ cell culture

Human CD34^+^ cells were cultured in hematopoietic stem cell expansion medium consisting of StemSpan SFEM II (StemCell Technologies, Cat: 09655) supplemented with 100 ng/ml human stem cell factor (SCF, Stemimmune LLC, Cat: HHM-SF-1000), 100 ng/ml human FMS-like trysine kinase 3 ligand (FLT3L, Stemimmune LLC, Cat: HHM-FT-1000), 50 ng/ml of human thrombopoietin (TPO, Stemimmune LLC, Cat: HHM-TP-0100), and 10 μg/ml of low-density lipoproteins (StemCell Technologies, Cat: 2698). Compounds were added in the HSC expansion medium with the indicated concentrations. 1 × 10^5^ CD34^+^ CB cells/ml were cultured in suspension with ultralow attachment six-well plates (Corning, Cat: 3471) at 37 °C and 5% CO_2_ in air. JNK-IN-8 (2 μM, Selleck, Cat: S4901) dissolved in DMSO (Sigma, Cat: D2650) was added in the HSC expansion medium and the culture medium was changed every other day. 0.01% DMSO was added as the control condition. Cell samples were collected at different days for transplantation, flow cytometry analysis and functional assay. Other compounds including SP600125 (5 μM, Selleck, Cat: S1460), Tanzisertib (2 μM, Selleck inhibitor, Cat: S8490), AEG3482 (10 μM, Tocris, Cat: 2651), TCS JNK 6o (10 μM, Tocris, Cat: 3222), JNK Inhibitor IX (0.1 μM, Selleck inhibitor, Cat: S7508) and BI-78D3 (2 μM, Selleck inhibitor, Cat: S8201) were used. Cultured cells were collected on indicated days for analysis and transplantation.

### Colony-forming unit (CFU) assay

Fresh CB CD34^+^ cells or cultured CD34^+^ cells were suspended in 200 μl IMDM (Thermo Fisher Scientific, Cat: 12440061) plus 2% FBS (Hyclone, Cat: SH30396), and then were inoculated in 2 ml methylcellulose-based medium (MethoCult™ H4435 Enriched, STEMCELLS Technologies, Cat: 04435) at 1,000 or 5,000 cells per well as indicated in ultralow attachment six-well plates (Corning, Cat: 3471). The plates were then placed in the incubator at 37 °C and 5% CO_2_ in air. The number of different CFU types including CFU-granulocyte (CFU-G), CFU-macrophage (CFU-M), CFU-granulocyte and macrophage (CFU-GM, CFU-erythrocyte (CFU-E), erythroid burst-forming unit (BFU-E), CFU-granulocyte, erythroid, macrophage, and megakaryocyte (CFU-GEMM) was counted 14 days later. Each assay was performed in triplicates.

### Flow cytometry analysis

Cultured cells were collected at different time and incubated with the indicated antibodies for 30 min at 4 °C in PBS containing 0.5% BSA (Sigma, Cat: A1470–100G). Next, the cells were washed three times with PBS and suspended in 0.2 ml PBS for analysis. Flow cytometry analysis was performed using FACSVerse (BD) or LSRFortessa (BD). The data were analyzed using FlowJo-V10 (BD). The following antibodies were used: FITC anti-human CD34 (Biolegend, Cat: 343604), PE anti-human CD38 (Biolegend, Cat: 356604), PE-Cy7 anti-human CD49f (Biolegend, Cat: 313622), APC anti-human CD90 (Biolegend, Cat: 328114), APC-Cy7 anti-human CD45RA (Biolegend, Cat: 304128). For analysis of engrafted human hematopoietic lineages, peripheral blood cells were collected at indicated time. BM was isolated at 21 weeks post transplantation, and cells were stained with following antibodies: PE-Cy7 anti-human CD45 (Biolegend, Cat: 304016), FITC anti-mouse CD45 (Biolegend, Cat: 103108), PE anti-human CD19 (Biolegend, Cat: 302208), APC anti-human CD33 (Biolegend, Cat: 366606).

### Primary transplantation and monitoring of human CB CD34^+^ cells in NPG mice

Male mice were sub-lethally irradiated 4–6 h before transplantation, and upon transplantation, they were anaesthetized by avertin. Fresh CD34^+^ CB cells or their progeny present in 10-day cultures were transplanted by intra-femorally with 29 G insulin syringe (BD, Cat: 320310). Mice recipients were placed back to IVC cage after regaining consciousness. Repopulated human cells in NPG mice peripheral blood (PB) and BM were monitored by flow cytometry at indicated time point posttransplantation. BM cells of NPG mice were collected by flushing the two femurs (at week 21 post transplantation) with PBS. The proportion of reconstituted human cells in PB was detected at 5, 9, 12, and 16 weeks posttransplantation and was analyzed referring to flow cytometry analysis protocol.

### Secondary transplantation and monitoring of human cells in vivo

Mice with average engraftment efficiency for the primary transplantation were selected for secondary transplantation from each group (JNK-IN-8, DMSO-expanded cells and uncultured cells). These mice were dislocated. The femur and the tibia were then collected, and the marrow was flushed out with PBS containing 0.5% BSA. BM cells were pipetted into single cells. Cells with different dosage were transplanted at 1 × 10^7^, 1 × 10^6^, 5 × 10^5^, and 1 × 10^5^ cells per female mouse, with five mice recipients for each dosage.

For detecting the reconstituting efficiency of secondary transplants, BM cells of the secondary mice were harvested and analyzed 21 weeks posttransplantation. Flow cytometry analysis was performed on freshly collected BM cells. Cells were washed by PBS with 0.5% BSA, and then stained with PE-Cy7 anti-human CD45 (Biolegend, Cat: 304016), FITC anti-mouse CD45 (Biolegend, Cat: 103108), PE anti-human CD19 (Biolegend, Cat: 302208), APC anti-human CD33 (Biolegend, Cat: 366606). Flow cytometry analysis was performed using LSRFortessa (BD).

The following is the correspondence between antibody labeling and cell populations: CD33 (myeloid cells); CD19 (lymphoid cells).

Since T-cell reconstitution correlates poorly with LT-HSC engraftment in other studies^31^ and may represent graft versus host disease, it was not monitored.

### Analysis of the engraftment efficiency from peripheral blood cells of transplanted mice

At different detecting time points including 5, 9, 12, 16 weeks, nearly 100 μl peripheral blood was collected from transplanted mice tail vein and the engraftment efficiency was monitored. Peripheral blood was centrifuged at 350 g for 5 min and cells were treated with 1X red blood cell (RBC) Lysis Buffer (Biolegend, Cat: 420301) and incubated on ice for 4–5 min with occasional shaking, stopped by diluting the lysis buffer with PBS. Then, cells were washed with PBS and stained with PE-Cy7 anti-human CD45 (Biolegend, Cat: 304016), FITC anti-mouse CD45 (Biolegend, Cat: 103108) PE anti-human CD19 (Biolegend, Cat: 302208), and APC anti-human CD33 (Biolegend, Cat: 366606). Flow cytometry analysis was performed using FACSVerse (BD).

### Plasmid

All inserts of shRNAs were designed and synthesized by Tsingke Company, and then cloned to SF-LV-shRNA-EGFP vector^32^. Sh-ctrl denotes scrambled shRNA, Sh-J1, and Sh-J2 denote shRNAs targeting c-Jun. Their sequences are shown below.

Sh-ctrl sequence:

5′-CAACAGAAGGCTCGATTCTCCGAACGTGTCACGTTTAGGCCTAACGTGACACGTTCGGAGAAAATTCGAGCAATTATCT-3′

Sh-J1 (1# c-Jun shRNA) sequence:

5′-CAACAGAAGGCTCGACAAACCTCAGCAACTTCAATTAGGCCTATTGAAGTTGCTGAGGTTTGAATTCGAGCAATTATCT-3′

Sh-J2 (2# c-Jun shRNA) sequence:

5′-CAACAGAAGGCTCGACCGGTAGTACTCCTTAAGAACACAACTCGAGTTGTGTTCTTAAGGAGTACTATTTTTGAATTCGAGCAATTATCT-3′

### Lentivirus packaging, concentration, and titration

293 T cells were cultured in Dulbecco’s modified Eagle’s medium (DMEM) (Gibco, Cat: 8117271) supplemented with 10% fetal bovine serum (FBS, Hyclone, Cat: SH30396), 1% penicillin/streptomycin (Gibco, Cat: 15140148), 1% NEAA (Gibco, Cat: 11140050), and 1% GlutaMAX (Gibco, Cat: 35050061). Lentivirus was packaged by the plasmids psPAX2 (Cat: 36052) and pMD2.G (Cat: 12259), which were purchased from Addgene. For lentivirus production, 8 × 10^6^ 293 T cells were plated in 10 ml media in a 10 cm tissue culture dish and incubated at 37 °C, in a 5% CO_2_ incubator overnight. Cells were transfected by the calcium phosphate co-precipitation method. In total, 12 μg lentiviral shRNA plasmid, 10 μg psPAX2 plasmid, and 3 μg pMD2.G plasmid were mixed in a 15 ml tube by vortex, and 50 μl 2.5 M CaCl_2_ solution was added into the mixture, sterile water was added to ensure the total volume of the liquid be 500 μl. Then the mixture was gently added dropwise into 500 μl 2xHBS (pH 7.12) in a tube. The slightly turbid mixture was vortexed for seven times, and then added to the cells in the culture dish dropwise. The culture dish was swirled to disperse the mixture evenly. Cells were incubated at 37 °C, in 5% CO_2_. After 12 h, the medium was changed. Viral supernatant was collected 36 h after changing the medium.

The medium recovered from 293 T cells supernatant was filtered with a 0.22-μm filter (Millipore, Cat: SLMP025SS) to remove cell debris. Viruses were then pelleted by spinning at 24,600 rpm for 90 min at 4 °C with Beckman ultraspeed centrifugal machine. Supernatant was abandoned and the virus pellet was suspended by cell culture medium (Stemspan medium supplemented with SCF, TPO, FLT3L). For titration, 293 T cells were passaged into 24-well plates for 2 × 10^4^ cells per well with DMEM supplemented with 10% FBS and incubated overnight at 37 °C, in 5% CO_2_. The next day, concentrated virus (0.001 μl, 0.01 μl, 0.1 μl) was added to each well with polybrene (8 ng/ml, Yeasen, Cat: 40804ES86). After incubating for 8–12 h, medium was aspirated and fresh medium was added. Three days later, cells were digested with Trypsin (Gibco, Cat: 25300–062) and collected by 1.5 ml tubes, and then analyzed by flow cytometry to detect the frequency of EGFP-positive cells. The concentrated viruses were about 10^8^ TU/ml.

### Viral transduction of CB CD34^+^ cells

The collected CD34^+^ cells were cultured with 2 × 10^5^ cells per well in 24-well plate at 37 °C, in 5% CO_2_ for 2 days. Cells were collected by centrifuge and supernatant was discarded. In total, 200 μl viruses and 200 μl culture medium supplemented with 8 μg/ml polybrene (Yeasen, Cat: 40804ES86) were gently mixed with cell pellet and added into each well. The culture plates were spun at 2,500 rpm for 90 min at 25 °C, and then placed back to incubator. After 12 h’ incubation at 37 °C, in 5% CO_2_, medium was changed. Three days later, EGFP^+^ frequency was monitored by flow cytometry.

### Transplantation of *shRNA-*transduced cells and monitoring of human cells in vivo

CD34^+^ cells were cultured for 2 days and was then transduced by two different shRNAs for c-Jun (shRNA-J1 and shRNA-J2). After 5-day culture, each of the shRNA-transduced cells were transplanted intravenously into five female mice at a dose of 5 × 10^5^ cells per mouse. Nineteen weeks posttransplantation, the mice were killed. The femur and the tibia were then collected and the marrow was flushed out with PBS containing 0.5% BSA. BM cells were pipetted into single cell and stained with PE-Cy7 anti-human CD45 (Biolegend, Cat: 304016) and APC anti-mouse CD45 (Biolegend, Cat: 103112). Flow cytometry analysis was performed using FACSVerse (BD).

### Quantitative real-time PCR

Total RNA of indicated cells was isolated from RNA isolation Kit (QIAGEN, Cat:74034) according to the manufacturer’s protocol. The Easy transcriptase kit (Transgene, Cat: AT311–03) was used for cDNA synthesis from total RNA. Quantitative real-time PCR was performed with a BIO-RAD CFX Connect™ Real-Time PCR Detection System (Bio-rad, Cat: 1855201) in duplicates from at least three biological samples. The quantitative PCR was carried out in a volume of 5 μl using the iTaq SYBR Green super-mix with Rox (Bio-Rad, Cat: 1725850). The PCR protocol was as follows: first, 95 °C for 10 min to activate the polymerase, followed by 40 cycles at 95 °C for 10 s (for denaturation), 60 °C for 10 s (for annealing), and 72 °C for 10 s (for extension). Values for mRNA expression were normalized to the expression of Actin. The primer sets for the detection of single genes are listed in Table S5.

### CFSE labeling

CD34^+^ CB cells (1 × 10^6^ cells/ml) were labeled with 5 μl carboxyfluorescein diacetate succinimidyl ester (Biolegend, Cat: 423801) according to the manufacture’s guidelines. Labeled cells were cultured for 24 h before they were stained with APC-labeled anti-human CD34 antibody (Biolegend, Cat: 343608) and sorted for CD34^+^CFSE^+^ cells using a FACS MoFlo (Beckman). Sorted cells were then resuspended in HSC expansion medium supplemented with DMSO (0.01%) or JNK-IN-8 (2 µM). Cell aliquots after 2 and 4 days in culture were stained with APC anti-human CD34 (Biolegend, Cat: 343608) and APC-Cy7 anti-human CD45RA antibody (Biolegend, Cat: 304128) before cells were analyzed for CFSE intensity by FCS express version 6 software.

### Limiting dilution analysis

The HSC frequency was quantified by extreme limiting dilution analysis^33^ (http://bioinf.wehi.edu.au/software/elda/), with 95% confidence intervals. We set > 1% human CD45^+^ in all CD45^+^ cells in the recipient BM 21 weeks posttransplantation as positive engraftment in our study. This criterion was applied to LDA calculation in both primary and secondary transplantation.

### Western blot

In total, 5 × 10^5^ fresh CD34^+^ cells were cultured with JNK-IN-8 (2 μM) or DMSO (v/v 0.01%) supplemented with 10% serum for 30 min, and then were pelleted and washed with PBS. The pellets were resuspended with 45 μl PBS and 15 μl lysis buffer (200 mM Tris-HCL, 8% SDS, 400 mM DTT, 0.1% bromophenol blue, 40% glycerol) and incubated at 100 °C for 10 min for lysis. The lysed solutions were electrophoresed in 10% SDS-PAGE and transferred onto PVDF membrane. The membranes were incubated with the appropriate primary antibodies (rabbit anti-total-c-Jun, Cell signaling, Cat:9165; rabbit anti-phosphor-c-Jun (ser63), Cell signaling, Cat: 9261; mouse anti-GAPDH, CWBIO, Cat: 0100 A) in 4% nonfat milk at 4 °C overnight. Then they were incubated with the horseradish peroxidase (HRP)-conjugated goat anti-mouse/rabbit IgG (Jackson Laboratories) as secondary antibodies and visualized by Tanon infrared imaging system.

### RNA-seq library preparation

Total RNA was isolated from day 7 JNK-IN-8 (2 μM), DMSO (v/v 0.01%), and only-cytokine (NC) cultured CB CD34^+^ cells using the RNeasy Plus Micro Kit (QIAGEN, 74034). RNA sequencing libraries were constructed using the NEB Next Ultra RNA Library Prep Kit for Illumina (NEB England BioLabs, E7530L). The fragmented paired-end libraries were sequenced using an Illumina HiSeq-PE150.

### Transcriptome analysis

The generated sequencing reads were mapped against human genome build hg19 by TopHat alignment software tools. The expression values for each sample were calculated by reading the bam file of aligned reads using cuffquant. The expression values were then merged into a single FPKM table by cuffnorm. For Fig. 5a, b, the JNK pathway gene list was downloaded from QIAGEN GeneGlobe Pathways database (https://www.qiagen.com/us/shop/genes-and-pathways/pathway-details/?pwid = 266). The gene’s relative expression was calculated in two steps: First, the fold change was calculated by dividing J8 sample by DMSO and NC replicates separately. And then, apply log base 2 transformation to the average fold change. The gene’s position on *x* axis was sorted by the absolute log2 (average fold change) value. The heatmap shown in Supplementary Fig. S3a was generated by R package pheatmap. The resource of R packages is as follows:

TopHat 2.1.1 (ref. ^34^) https://ccb.jhu.edu/software/tophat/index.shtml;

Bowtie 2.2.9 (ref. ^35^) http://bowtie-bio.sourceforge.net/bowtie2/index.shtml;

samtools 0.1.18 (ref. ^36^) http://samtools.sourceforge.net;

Cufflinks 2.2.1 (ref. ^37^) https://github.com/cole-trapnell-lab/cufflinks;

R 3.5.0 https://www.r-project.org;

ggplot2 2.2.1 http://ggplot2.tidyverse.org;

pheatmap  1.0.8 https://github.com/raivokolde/pheatmap.

### Quantification and statistical analysis

Statistical analysis was performed with GraphPad Prism software. Data are shown as the mean with standard deviation (SD). Pairwise comparisons between different groups were assessed using unpaired *t* test. For all analyses, *p* < 0.05 was considered statistically significant. The statistical significance, and *n* value are reported in the Figure legends. All the flow analysis data except for CFSE labeling monitoring were processed with FlowJo v10 software. All the Figures were prepared by Adobe Illustrator.

## Electronic supplementary material

Supplementary Information

## Data Availability

All software used in this study is described in the methods sections and available online. The RNA-seq data has been deposited and the accession number is GSE114524.

## Electronic supplementary material

**Supplementary Information** accompanies the paper at (10.1038/s41421-018-0072-8).
