# Supplementary material for: Targeting JNK pathway promotes human hematopoietic stem cell expansion
Source: Cell Discov. 2019 Jan 8;5:2. doi: 10.1038/s41421-018-0072-8 (PMC6323118; doi:10.1038/s41421-018-0072-8)
Supplement: Supplementary file 1 — Supplementary Information [file 41421_2018_72_MOESM1_ESM.pdf]

Supplementary Figures

Fig.S1: Summary of JNK-IN-8 effect on HSPC expansion, related to Fig.1 and Fig.2

Fig.S2: Summary of lineage distribution of repopulated cells in primary recipients, related to Fig.3.

Fig.S3: Summary of c-Jun inhibition on HSPC expansion, related to Fig.5.

Supplementary Tables

Table S1. Effect of J8 on enriched phenotypic hematopoietic stem and progenitor cell populations after 10 days in culture (20, 000 initiating cells were cultured), related to Fig. 1 and Fig. S1.

Table S2. Effect of J8 on CB-derived $CD34^+$ hematopoietic stem and progenitor cell expansion on day 10, related to Fig. 2.

Table S3. Summary of human engraftment in the BM of NPG recipients, related to Fig. 3, Fig.4 and Fig. S2. (see supplemental excel files)

Table S3 (A). Summary of human engraftment in primary NPG recipient BM at 21 weeks post transplantation, related to Fig. 3 and Fig. S2.

Table S3 (B). Summary of human engraftment in secondary NPG recipient BM at 21 weeks post transplantation, related to Fig. 4.

Table S4. Effect of c-Jun knockdown on CB-derived $CD34^+$ hematopoietic stem and progenitor cell expansion, related to Fig.5 and Fig. S3.

Table S4 (A). Summary of effect of *c-Jun* knockdown on CFUs, related to Fig.5.

Table S4 (B). Summary of effect of *c-Jun* knockdown on engraftment 19 weeks post transplantation, related to Fig.S3.

Table S5. List for qPCR primers, related to Material and methods.

Fig. S1

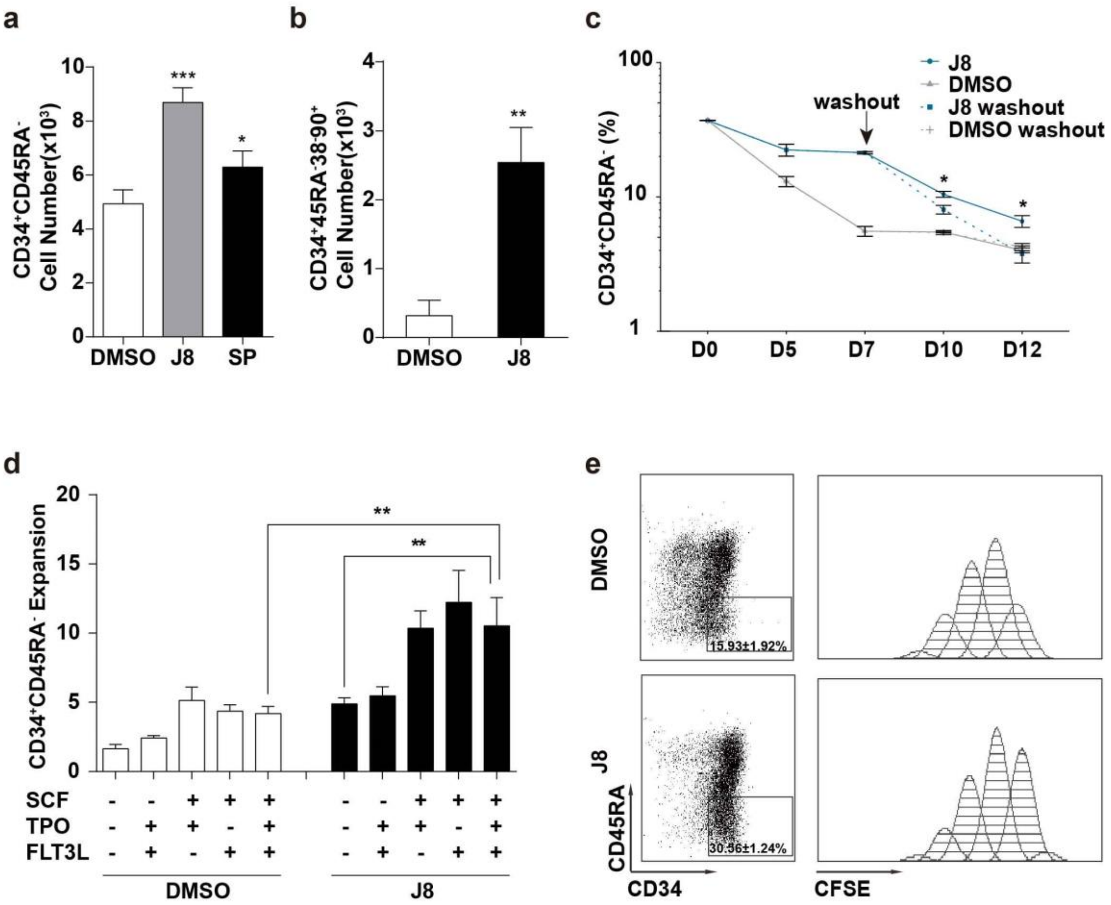

Fig.S1: Summary of JNK-IN-8 effect on HSPC expansion, related to Fig.1 and Fig.2

a) Cell number of $CD34^+CD45RA^-$ subsets after 7-day culture of 100,000 fresh $CD34^+$ cells in the presence of DMSO, J8 (2μM) or SP600125 (5μM) (n=3 experiments).

b) Cell number of $CD34^+CD45RA^-CD38^-CD90^+$ subsets after 10-day culture of 10,000 fresh $CD34^+$ cells in the presence of DMSO or J8 (2μM) (n=3 experiments).

c) Change of $CD34^+CD45RA^-$ population frequency after washout of J8 (2μM) or DMSO on day 7 $CD34^+$ cells which were cultured for further 5 days (n=3 experiments) (This data was drawn by R).

d) $CD34^+CD45RA^-$ population expansion calculated on day 7 in $CD34^+$ cells cultured with J8 (2μM) or DMSO in the serum free medium in the absence or presence of indicated cytokines (n=3 experiments).

e) Proliferation of DMSO or J8-cultured $CD34^+$ cells, characterized by flow cytometry on day 4 after CFSE labelling.

All data shown as mean values ± SD. Statistical significance was assessed using unpaired t-test, where *$p < 0.05$ and **$p < 0.01$. See also Supplementary Table S2.

Fig. S2

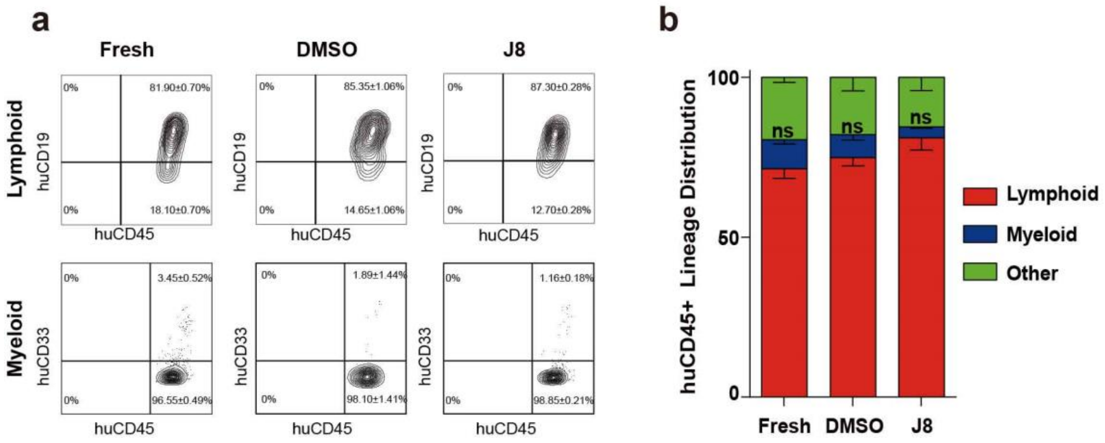

Fig.S2: Summary of lineage distribution of repopulated cells in primary recipients, related to Fig.3.

a) Representative flow plots showing myeloid ($CD33^+$) and lymphoid ($CD19^+$) reconstitution of fresh-isolated, DMSO and J8-cultured $CD34^+$ cells in the BM of primary recipients 21 weeks post transplantation.

b) Lineage distribution of human $CD45^+$ cells in the primary recipient mice BM 21 weeks after transplantation among fresh, DMSO and J8-cultured $CD34^+$ cells. Lymphoid, $CD19^+$ cells; Myeloid, $CD33^+$ cells; Other, cell population other than $CD19^+$ and $CD33^+$ cells. ns, not significant. Details see Supplementary Table S3(A). All data shown as mean values ± SD. Statistical significance was assessed using unpaired t-test.

Fig. S3

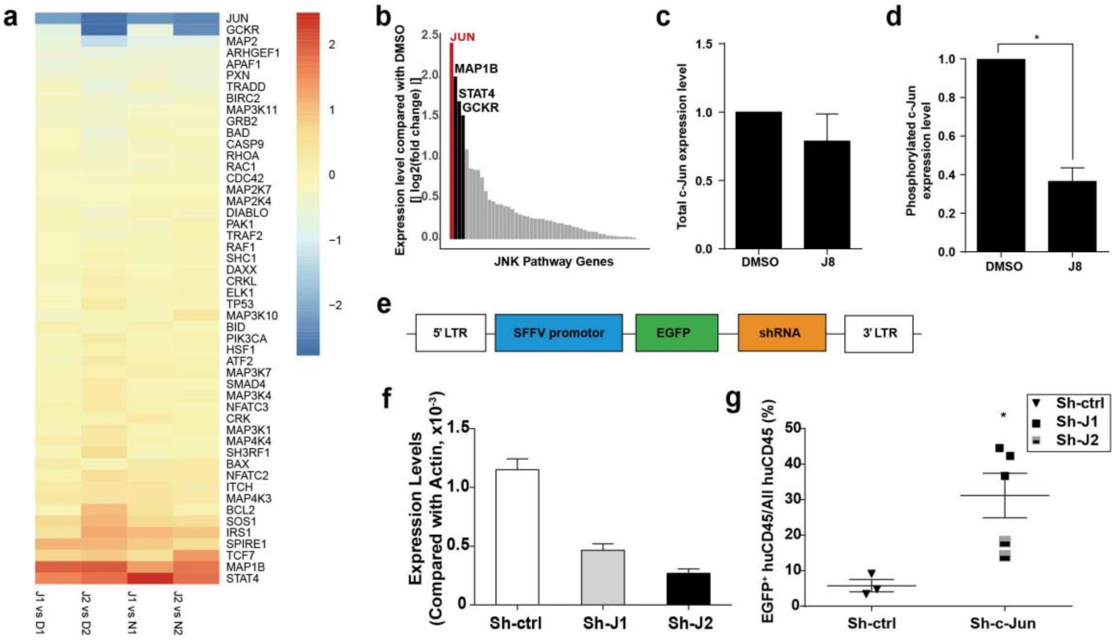

Fig.S3: Summary of c-Jun inhibition on HSPC expansion, related to Fig.5.

a) Heat map showing JNK pathway genes' relative expression of $CD34^+$ cells after 7-day culture supplemented with J8 (J1 and J2 indicate 2 replicates of J8-treated cells), DMSO (D1 and D2 indicate 2 replicates of DMSO-treated cells) or only cytokines (N1 and N2 indicate 2 replicates of cytokines-only-cultured cells) (This data was drawn by R).

b) The absolute value of the log2 change ratio of JNK pathway gene expression level of J8-treated day7 $CD34^+$ cells compared with DMSO-treated day7 $CD34^+$ cells (This data was drawn by R).

c) Quantification of relative protein expression of total c-Jun in $CD34^+$ cells cultured with DMSO or J8 following serum stimulation for 30 minutes (n=3 experiments, p=0.2046).

d) Quantification of relative protein expression of phosphorylated c-Jun for DMSO and J8-treated $CD34^+$ cells following serum stimulation for 30 minutes. (n=3 experiments, p=0.0117)

e) Schematic view of *c-Jun* shRNA construct.

f) Relative *c-Jun* mRNA expression of indicated transduced $CD34^+$ cells on day 5 after transduction (n=2 experiments).

g) $EGFP^+$ cell frequency in total human $CD45^+$ cell population in the PB of mice recipients that injected with scrambled shRNA-transduced (Sh-ctrl) or c-Jun shRNA-transduced (Sh-c-Jun) 50,000 day 5 $CD34^+$ cells 19 weeks after transplantation (n=5 mice from two independent experiments). See also Table S4B.

Sh-ctrl, $CD34^+$ cells transduced with scrambled shRNA; Sh-J1, $CD34^+$ cells transduced with 1# c-Jun shRNA; Sh-J2, $CD34^+$ cells transduced with 2# c-Jun shRNA. See also Supplementary Table S4 (B).

All data shown as mean values ± SD. Statistical significance was assessed using unpaired t-test, where *$p < 0.05$.

Table S1. Effect of J8 on enriched phenotypic hematopoietic stem and progenitor cell populations after 10 days in culture (20, 000 initiating cells were cultured), related to Fig. 1 and Fig. S1.

Concentration	Total cells ($x10^4$)	$CD34^+$ ($x10^4$)	$CD34^+CD45RA^-$ ($x10^{4)}$	$CD34^+CD45RA^-CD38^-CD90^+$ ($x10^4$)	$CD34^+CD45RA^-CD38^-CD90^+CD49f^+$ ($x10^4$)
0	184.5	82.82131	7.255147	0.088513	0.088513
0.1μM	142	71.13575	5.697974	0.060968	0.060968
0.2μM	187.5	89.4345	7.637706	0.33835	0.230755
0.5μM	180	101.2176	9.585307	0.461053	0.282626
1μM	158.5	88.84099	8.271096	0.354003	0.177001
1.2μM	150	87.0861	9.405299	0.298148	0.161596
1.4μM	169.5	97.96761	13.9114	0.93902	0.408474
1.5μM	153.5	93.00258	12.64835	0.155575	0.144529
1.6μM	145	92.97603	12.83069	0.677461	0.399024
1.8μM	110	68.14456	10.22168	0.627611	0.226568
2μM	150.5	94.1528	17.22996	0.404904	0.309752
2.5μM	139.5	80.50908	11.5128	0.484689	0.231681
3μM	100	63.4382	14.7811	0.6415	0.34641
4μM	69.5	43.94777	13.49197	0.348093	0.246102
5μM	52	30.72753	13.3972	0.377801	0.302996
10μM	11	5.085806	3.071827	0.078639	0.063697

Table S2. Effect of J8 on CB-derived CD34$^+$ hematopoietic stem and progenitor cell expansion on day 10, related to Fig. 2.

**Colony type**	**CFU/1,000 fresh CD34$^+$ cells**	**CFU frequency/1,000 cells on day 10**		**Fold increase (J8 VS. DMSO)**
		DMSO cultured cells	J8 cultured cells	
G	46.0±13.0	29.8±7.4	19.4±10.1	0.7
M	18.7±6.1	158.3±60	89.4±55.8	0.6
GM	98.3±2.4	67.7±6.6	102.5±18.3	1.5
GEMM	5.3±0.5	12.6±17.8	57.0±27.6	4.5
BFU-E	43±5.1	7.7±8.0	19.1±9.8	2.5
CFU-E	38.3±2.4	28.9±3.0	33.3±7.5	1.2
Total	249.7±11.6	50.0±9.9	60.2±8.9	1.2

Table S3. Summary of human engraftment in the BM of NPG recipients, related to Fig. 3, Fig.4 and Fig. S2. (see supplemental excel files)

Table S3 (A). Summary of human engraftment in primary NPG recipient BM at 21 weeks post transplantation, related to Fig. 3 and Fig. S2.

Table S3 (B). Summary of human engraftment in secondary NPG recipient BM at 21 weeks post transplantation, related to Fig. 4.

Table S3 (A) Summary of human engraftment in primary NPG recipients BM at 21 weeks post transplantation, related to Fig. 3 and Fig. S2.

mouse ID	injection dose of day 0 equivalent CD34+ cells	CD45 (%)	B cell (%)	Myeloid cell (%)
0630 *O1 2#	10000	46.9	87.9	1.65
0630 O1 3#	10000	62.6	84.8	4.69
0630 O1 4#	10000	43.5	86.6	1.45
0630 O2 1#	2000	35.2	88.3	1
0630 O2 2#	2000	25.1	86.2	3.73
0630 O2 3#	2000	12.6	89.8	2.02
0630 O2 4#	2000	25.7	87.7	0.5
0630 O2 5#	2000	0.96	18.5	0
0630 O3 1#	200	0.58	0.93	2.8
0630 O3 2#	200	0.58	0	0
0630 O3 3#	200	0.33	0	1.25
0630 O3 4#	200	1.02	0	0
0701 *J1 1#	10000	22.2	88.4	0.94
0701 J1 4#	10000	31.1	85.6	2.82
0701 J1 5#	10000	18.2	91	2.97
0710 J1 1#	10000	14.6	76	6.43
0710 J1 2#	10000	27.4	38.5	41
0710 J1 3#	10000	43.1	90.1	1.42
0701 J2 1#	2000	2.23	0.39	1.17
0701 J2 2#	2000	11.5	86.9	1.13
0701 J2 3#	2000	30	91.5	0.58
0701 J2 4#	2000	46.6	92	0.73
0710 J2 1#	2000	5.57	82.2	7.95
0710 J2 2#	2000	3.47	84.4	6.47
0710 J2 3#	2000	16	92.5	2.04
0701 J3 1#	200	1.28	0.52	1.55
0701 J3 2#	200	0.93	2.54	2.54
0701 J3 3#	200	1.29	0.7	0
0701 J3 4#	200	0.37	1.69	6.78
0710 J3 1#	200	1.17	70.3	5.07
0710 J3 2#	200	1.03	85.7	7.45
0710 J3 3#	200	0.6	0	0
0710 J3 4#	200	0.35	0	0
0701 *N1 2#	10000	14.3	89.5	1.93
0701 N1 3#	10000	19.8	89.5	2.37
0701 N1 5#	10000	9.14	76.7	12.4
0710 N1 1#	10000	8.49	83.9	5.51
0710 N1 2#	10000	0.44	52.8	33.3
0710 N1 3#	10000	29.4	74.1	5.61
0701 N2 1#	2000	2.42	50.2	4.68
0701 N2 2#	2000	2.84	30.6	0.7
0701 N2 3#	2000	1.46	4.58	12.2
0710 N2 1#	2000	13.1	12.6	1.93
0710 N2 2#	2000	44.9	0	0.54
0710 N2 3#	2000	8.9	0	0
0701 N3 1#	200	1.21	0	33.3
0701 N3 2#	200	1.42	0.72	25.9
0710 N3 1#	200	0.34	0	3.77
0710 N3 3#	200	0.13	0	31.1
0710 N3 4#	200	1.31	77.7	8.03

*. All "J" denotes for mice that received CD34+ cells treated with J8 (2μM) plus cytokines;
all "N" denotes for mice that received CD34+ cells treated with DMSO plus cytokines;
all "O" denotes for mice that received freshly-isolated CD34+ cells

The value of the confidence choice entered was "0.95'
The value of the observed choice is "TRUE"
The value of the test_unit_slope choice is "TRUE"
The value of the test_difference choice is "TRUE"

Limiting dilution data entered.

Counter	Dose	Tested	Response	Group
1	10000	6	6	J8
2	2000	7	7	J8
3	200	8	4	J8
4	10000	6	5	DMSO
5	2000	6	6	DMSO
6	200	5	3	DMSO
7	10000	3	3	Fresh
8	2000	5	4	Fresh
9	200	4	1	Fresh

The number of lines of data entered = 9

Confidence intervals for
1/(stem cell frequency)

Group	Lower	Estimate	Upper
DMSO	4172	1712	703
Fresh	2999	1111	412
J8	744	286	110

Overall test for differences in stem cell frequencies between any of the groups

Chisq	DF	P.value
9.08	2	0.0107

Pairwise tests for differences in stem cell frequencies

Group 1	Group 2	Chisq	DF	Pr(>Chisq)
DMSO	Fresh	0.509	1	0.475
DMSO	J8	9.07	1	0.0026
Fresh	J8	3.9	1	0.0482

Goodness of fit tests. These test whether the log-dose slope equals 1.
Rejection of the tests may be due either to batch effects (heterogeneity in the stem cell frequencies or assay success rate) or
Estimated slope is 0.43

Test	Chisq	DF	P Value
Likelihood	11	1	0.000898
Score test of	63	1	2.04E-15

Table S3 (B) Summary of human engraftment in secondary NPG recipient BM at 21 weeks post transplantation, related to Fig.4.

mouse ID	injection dose of total cells in primary recipients' BM	injection dose of human CD34+ cells in primary recipients' BM	CD45 (%)
1128 *J1-1	1x10^7	707000	14.3
1128 J1-2	1x10^7	707000	2.18
1128 J1-3	1x10^7	707000	23.7
1128 J1-5	1x10^7	707000	60.2
1128 J2-1	1x10^6	70700	0.79
1128 J2-2	1x10^6	70700	0.29
1128 J2-3	1x10^6	70700	0.77
1128 J2-4	1x10^6	70700	2.77
1128 J2-5	1x10^6	70700	1.7
1128 J3-2	5x10^5	35350	0.47
1128 J3-3	5x10^5	35350	1.44
1128 J3-4	5x10^5	35350	1.02
1128 J3-5	5x10^5	35350	0.36
1128 J4-1	1x10^5	7070	0.65
1128 J4-2	1x10^5	7070	1.19
1128 J4-3	1x10^5	7070	0.73
1128 J4-4	1x10^5	7070	1.25
1128 *N1-1	1x10^7	365000	0.99
1128 N1-2	1x10^7	365000	2.05
1128 N1-3	1x10^7	365000	5.24
1128 N2-1	1x10^6	36500	0.88
1128 N3-1	5x10^5	18250	0.72
1128 N3-2	5x10^5	18250	0.83
1128 N4-1	1x10^5	3650	0.53

*, All "J" denotes for mice that received bone marrow (BM) engrafts of CD34+ cells treated with J8 (2μM) plus cytokines from primary recipients;
all "N" denotes for mice tha received BM engrafts of CD34+ cells treated with DMSO plus cytokines from primary recipients

The value of the confidence choice entered was "0.95"
The value of the observed choice is "TRUE"
The value of the test_unit_slope choice is "TRUE'
The value of the test_difference choice is "TRUE'

Limiting dilution data entered.

Counter	Dose	Tested	Response	Group
1	707000	4	4	J8
2	70700	5	2	J8
3	35350	4	2	J8
4	7070	4	2	J8
5	365000	3	2	DMSO
6	36500	1	0	DMSO
7	18250	2	0	DMSO
8	3650	1	0	DMSO

The number of lines of data entered = 8

Confidence intervals for
1/(stem cell frequency)

Group	Lower	Estimate	Upper
DMSO	1536125	374111	91112
J8	157372	65714	27440

Overall test for differences in stem cell frequencies between any of the groups

Chisq	DF	P.value
5.02	1	0.0251

Goodness of fit tests. These test whether the log-dose slope equals 1.
Rejection of the tests may be due either to batch effects (heterogeneity in the stem cell frequencies or assay success rate) or to a failure of the stem cell hypothesis.
Estimated slope is 0.543

Test	Chisq	DF	P Value
Likelihood ratio test of single-hit model	2.07	1	0.15
Score test of heterogeneity	0.101	1	0.75

Table S4. Effect of c-Jun knockdown on CB-derived CD34$^+$ hematopoietic stem and progenitor cell expansion, related to Fig.5 and Fig. S3.

Table S4 (A). Summary of effect of *c-Jun* knockdown on CFUs, related to Fig.5.

**Colony type**	**CFU frequency/5,000 cells on day 5 after transduction**		
	Scrambled (Sh-ctrl)	*c-Jun* knockdown cells	
		Sh-J1	Sh-J2
G	2.3±3.3	0.7±0.9	0.7±0.9
M	26.3±5.4	13.7±5.2	10.3±0.5
GM	112.3±81.3	224.3±6.2	200.3±11.3
BFU-E	0.0±0.0	18.3±21.0	1.7±1.2
CFU-E	2.0±1.4	42.7±30.0	31.7±1.2
GEMM	0.0±0.0	1.7±0.9	1.3±0.5

Table S4 (B). Summary of effect of *c-Jun* knockdown on engraftment 19 weeks post transplantation, related to Fig.S3.

mouse ID	GFP$^+$CD45$^+$/ All huCD45$^+$ (%)
1226 * SC1-2	11.6
1226 SC1-4	3.4
1226 SC1-5	2.39
1226 * SJ1-1	33.9
1226 SJ1-2	30.2
1226 SJ1-3	35
1226 * SJ2-4	11.6
1226 SJ2-5	14.1

*: All "SC1" denotes mice injected with CD34$^+$ cells transduced with scrambled shRNA (sh-ctrl); all "SJ1" and "SJ2" denotes mice injected with CD34$^+$ cells transduced with shRNA targeting *c-Jun* (sh-J1 and sh-J2, respectively).

Table S5. List for qPCR primers, related to Material and methods.

*β-Actin*_ Forward	GACAGCAGTCGGTTGGAGCG
*β-Actin*_ Reverse	GGGACTTCCTGTAACAACGCATC
*c-Jun*_ Forward	GTGACGGACTGTTCTATGACT
c-Jun _ Reverse	GGGTTACTGTAGCCATAAGGT
*JUNB*_ Forward	ACGACTCATACACAGCTACGG
*JUNB*_ Reverse	GCTCGGTTTCAGGAGTTTGTAGT
*JUND*_ Forward	TCATCATCCAGTCCAACGGG
*JUND*_ Reverse	TTCTGCTTGTGTAAATCCTCCAG
*JNK1*_ Forward	TGTGTGGAATCAAGCACCTTC
*JNK1*_ Reverse	AGGCGTCATCATAAAACTCGTTC
*JNK2*_ Forward	GAAACTAAGCCGTCCTTTTCAGA
*JNK2*_ Reverse	TCCAGCTCCATGTGAATAACCT
